# Understanding “green” multicellularity: do seaweeds hold the key?

**DOI:** 10.3389/fpls.2014.00737

**Published:** 2015-01-20

**Authors:** Juliet C. Coates, Umm-E-Aiman, Bénédicte Charrier

**Affiliations:** ^1^School of Biosciences, University of BirminghamBirmingham, UK; ^2^Integrative Biology of Marine Models, CNRS, Station BiologiqueRoscoff, France; ^3^Integrative Biology of Marine Models, Sorbonne Universités, Université Pierre et Marie Curie Univ., Station BiologiqueRoscoff, France

**Keywords:** multicellularity, transitions, life-cycles, toolkit, macroalgae, plants

## Introduction

Living organisms are unicellular, composed of a single cell, or multicellular, where a group of up to ~10^12^ cells functions co-operatively (Kaiser, 2001). All multicellular organisms evolved from single-celled ancestors; every individual organism arises from a unicell and reproduces by forming unicells. Multicellularity enables competitive advantages, and may have shaped our oxygen-rich atmosphere (Grosberg and Strathmann, 1998; Kaiser, 2001; Schirrmeister et al., 2013). Multicellularity has evolved multiple times: animals, plants, algae, amoebae, fungi, and bacteria are or can all be multicellular (King, 2004; Grosberg and Strathmann, 2007; Rokas, 2008; Claessen et al., 2014). Multicellularity can be clonal (arising from division of a single cell) or aggregative (aggregation of genetically diverse cells), with clonal multicellularity considered evolutionarily more stable (Grosberg and Strathmann, 1998). The molecular mechanisms by which organisms become multicellular are not well understood. In this article, we outline eukaryotic multicellular evolution, and discuss how to increase our future understanding.

## Evolution of unicellular–multicellular transitions: a genetic toolkit for multicellularity?

The most well-studied group of multicellular organisms are animals, where multicellularity likely arose once, giving rise to today's diversity of complex forms. Organisms in animal sister lineages, the aquatic “protist” choanoflagellates and filastereans (forming holozoans collectively with animals), can be unicellular or multicellular. Comparison of unicellular holozoan and animal genomes suggests that part of the “toolkit” of genes required to orchestrate multicellular development (genes for cell adhesion, cell–cell signaling and certain transcription factors) was already present in unicellular ancestors of both holozoans and animals (Abedin and King, 2008; King et al., 2008; Sebe-Pedros et al., 2011; Fairclough et al., 2013; Suga et al., 2013), although “metazoan-specific innovations” also exist (Suga et al., 2013).

Data from algae extends this “common toolkit” hypothesis to other kingdoms. The *Chlamydomonas* (unicellular) and *Volvox* (simple multicellular) genomes are remarkably similar (Merchant et al., 2007; Prochnik et al., 2010), with very few species-specific genes, and expansion of *Volvox* extracellular matrix (ECM) gene families (Prochnik et al., 2010). Much is now understood about the evolutionary steps to multicellularity in Volvocine algae (Herron et al., 2009), but the underlying molecular–genetic mechanisms are unknown.

The genome sequence of *Ectocarpus*, a multicellular brown alga, reveals no obvious trends of specific gene loss/gain in independent multicellular lineages (Cock et al., 2010). *Ectocarpus* contains possible integrin domains, which are important for animal development and also present in unicellular Holozoa (Cock et al., 2010). *Ectocarpus* also highlights the independent evolution of large receptor-kinase protein families as a step to drive complex multicellular evolution from a eukaryotic ancestor (Cock et al., 2010), as also suggested in plants (Shiu and Bleecker, 2001) and holozoans (Hunter and Plowman, 1997; Suga et al., 2014). Multicellular fungi possess unique non-receptor kinases (Stajich et al., 2010). Thus, transitions to multicellularity most likely largely require co-option of pre-existing genes, via changes in expression or regulation.

## Understanding unicellular–multicellular life-cycle transition mechanisms

**Figure 1:**
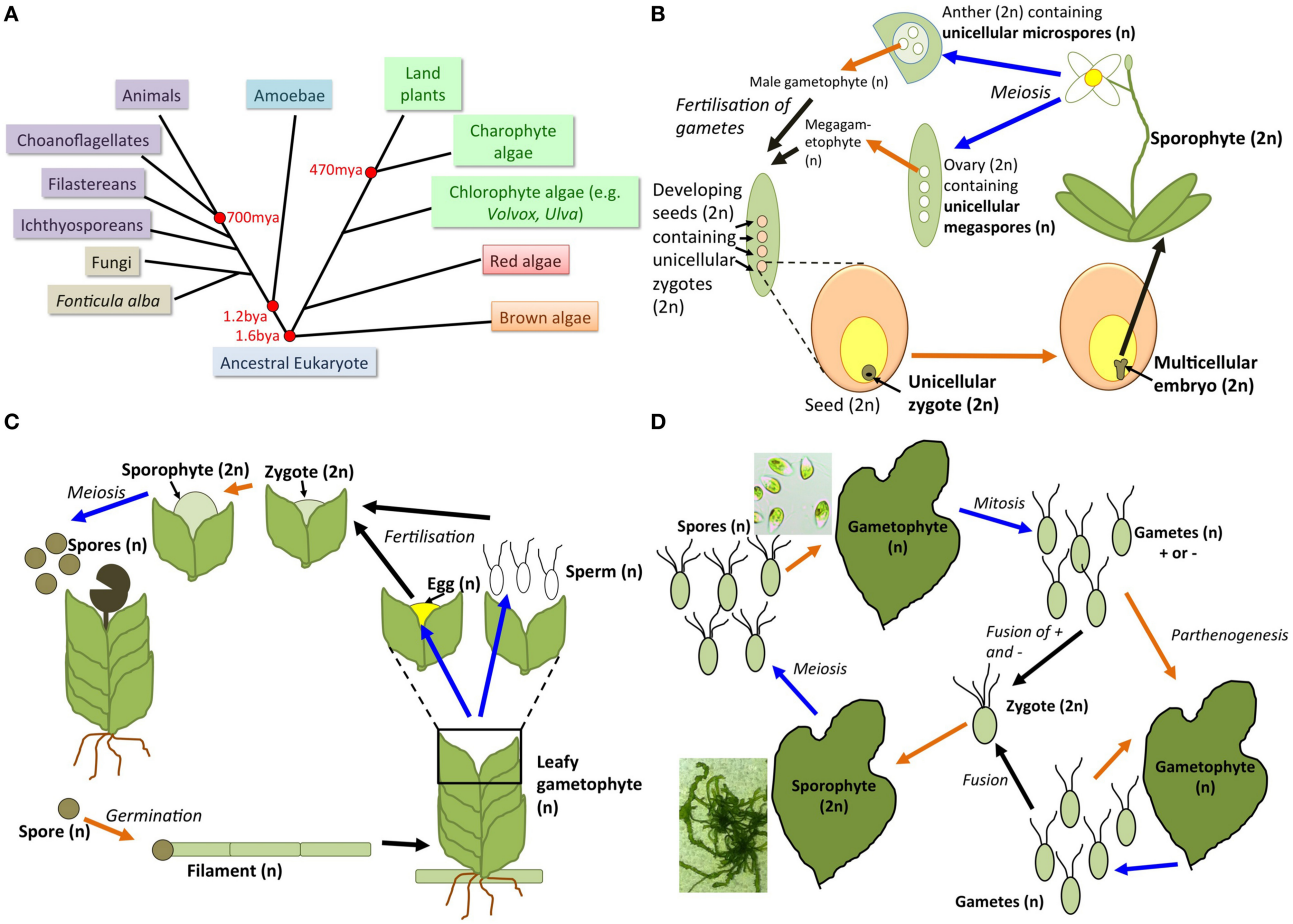
**Transitions between unicellular and multicellular states in plants, algae, and their relatives. (A)** Simplified tree of life showing the Unikont and plant/algal lineages and their evolutionary relationships, including divergence times in millions or billions of years ago (mya or bya, respectively). Animals, choanoflagellates, filastereans, and ichthyosporeans are collectively known as holozoans (purple boxes). Plants and green algae (green boxes) together with red algae (red box) form the Archaeplastida, while brown algae are part of a separate lineage evolving from a common eukaryotic ancestor 1.6 billion years ago. **(B)** Highly simplified flowering plant life cycle showing unicellular–multicellular transitions (orange arrow) and multicellular–unicellular transitions (blue arrows). The multicellular seed (orange) houses the unicellular diploid zygote (brown) surrounded by multicellular endosperm tissue (yellow). The zygote develops into a multicellular embryo within the seed, which then germinates to form the multicellular diploid adult sporophyte plant. Within the sporophyte flower, meiosis occurs to produce single-celled haploid gametes (female ovules and male pollen) in the multicellular diploid ovaries and anthers, respectively. The unicellular megaspores develop into a multicellular (seven-celled) haploid megagametophyte, completely surrounded by maternal multicellular tissue, prior to fertilization of one megagametophyte nucleus to form a zygote buried within the maternal- and developing seed-tissue. The microspores within the anther form a pollen grain with two cells that degenerate when the pollen germinates, allowing transfer of nuclei to the megagametophyte. Although the mature pollen grain (haploid gametophyte) is an accessible unicell it does not germinate to form a multicellular structure by itself, but only after fertilization has occurred. **(C)** Simplified life cycle of an early-evolving spore-bearing land plant, such as a moss (Bryophyte), showing unicellular–multicellular transitions (orange arrow), and multicellular–unicellular transitions (blue arrows). A haploid spore (brown) germinates to form a haploid multicellular filament, which eventually produces haploid multicellular leafy structures (gametophytes). Gametophytes produce haploid sperm or eggs, which fuse to form a unicellular diploid zygote, which divides by mitosis to form a multicellular diploid sporophyte. The sporophyte matures and then divides by meiosis to form haploid spores, which are released from the capsule. **(D)** Simplified life cycle of a macroalga such as *Ulva* (green seaweed) showing unicellular–multicellular transitions (orange arrows) and multicellular–unicellular transitions (blue arrows). Multicellular blade (thallus) tissue, a haploid gametophyte or a diploid sporophyte, arises from haploid spores and a diploid zygote, respectively. Gametes from two different mating types (+ and −) are required for fusion and zygote formation to occur. Sporophyte and gametophyte blades are essentially morphologically identical, as are gametes of different mating types. Isolated gametes in culture are capable of undergoing a parthenogenetic life cycle. Representative images of *Ulva linza* sporophyte thalli and spores in culture are shown (courtesy of Eleanor Vesty). Life cycles for red and brown algae are similar, but often more complex, with non-isomorphic multicellular stages and/or gametophyte or sporophyte reduced in size.

All multicellular organisms possess a unicellular life-cycle stage, undergoing a unicellular–multicellular transition in every generation. In the most complex organisms (animals and terrestrial plants), these transitions are challenging to characterize experimentally, as the unicells (gametes, zygotes) are hidden deep within host tissues (e.g., Figure 1B). However, there are eukaryotes of varying complexity that offer tractable systems to define molecular changes underpinning unicellular–multicellular transitions, enabling new opportunities for characterization and comparison.

### Modes of multicellularity on the Unikont branch

Animals, Holozoa, fungi, and amoebae (collectively, Unikonts) are a key branch of the tree of life (Figure 1A). Multicellularity has arisen many times in Unikonts, but there is no correlation between an organism's relatedness to animals and its mode of multicellularity. Several Holozoa have recently-identified multicellular life-cycle stages (Fairclough et al., 2010; Dayel et al., 2011; Sebe-Pedros et al., 2013; Suga and Ruiz-Trillo, 2013), with the ichthyosporean *Creolimax fragrantissima* forming a colony via a multinucleate syncitium (Suga and Ruiz-Trillo, 2013). The choanoflagellate *Salpingoeca rosetta* has clonal multicellularity, like animals (Fairclough et al., 2010), with two distinct multicellular morphologies, chains and rosettes (Dayel et al., 2011). Cytokinesis genes, cell–cell adhesion genes and receptor tyrosine kinases were all upregulated in rosettes (Fairclough et al., 2013), while a C-type lectin gene is required for rosette formation (Levin et al., 2014). The Filasterian *Capsaspora owczarzaki* assumes aggregative multicellularity, with deposition of ECM (Sebe-Pedros et al., 2013). *C. owczarzaki* upregulates integrin-mediated adhesion and signaling genes in aggregates compared to unicellular stages (Sebe-Pedros et al., 2013), strengthening the idea that multicellularity requires changes in gene regulation.

Reverse transitions from multicellularity likely occurred in Unikonts: fungi may have evolved as filamentous organisms >1 billion years ago (Butterfield, 2005), although many extant yeast species are entirely unicellular. Multicellularity can be selected for in the lab in the normally unicellular baker's yeast *Saccharomyces cerevisiae* (Ratcliff et al., 2012): this is clonal multicellularity, like in choanoflagellates and animals. Multicellular social amoebae diverged from Metazoa ~1.2 billion years ago (Figure 1A). The best-known example, *Dictyostelium*, forms aggregates from motile cells (Coates and Harwood, 2001; Schaap, 2011). Notably, the protist *Fonticula alba*, in the sister group to fungi, also shows aggregative multicellularity (Brown et al., 2009). Unicellular–multicellular transcriptional changes are conserved over large evolutionary distances: *Dictyostelium discoideum* and *Dictyostelium purpureum* diverged ~400 MYA but share similar morphologies and incredibly similar global gene expression profiles during their unicellular–multicellular transitions (Parikh et al., 2010).

### Multicellularity and environmental sensing

Unicellular–multicellular changes play key roles in the adaptation of some microorganisms to the environment. Selection pressures driving transitions to multicellularity include nutrient stress, predation and competition. Social amoebae aggregate in response to starvation, forming a fruiting body containing stress-resistant spores (Schaap, 2011). Some strains of *S. cerevisiae* form filaments in response to nitrogen starvation (Liu et al., 1996). Some green microalgae (including *Desmodesmus* and *Scenedesmus*) transition between unicellular and multicellular states, similarly to choanoflagellates (Lürling, 2003). These algae form multicellular structures due to nutrient-limitation (Trainor and Shubert, 1974; Siver and Trainor, 1981) competitor algae (Leflaive et al., 2008), animal predators (Hessen and Van Donk, 1993; Lampert et al., 1994; Lürling and Van Donk, 1996; Kampe et al., 2007) or toxins (Lürling, 2003).

Becoming multicellular to escape predators can have a physiological cost via a reduction in growth rate. The benefit of multicellularity as a defense mechanism depends on the population dynamics of co-occurring species and the environmental conditions determining the prey's growth rate: in the absence of a predator and under nutrient-replete competitive conditions multicellularity can have an adverse effect on the organism's fitness (Yokota and Sterner, 2011). Multicellularity can also affect green organisms' productivity: in *Desmodesmus armatus*, light-acclimation occurred in two-celled colonies, whereas maximum photosynthetic rates occurred in four-celled colonies (Matusiak-Mikulin et al., 2006). As microalgal multicellular morphs and unicells occupy different spaces in the water, unicellular–multicellular transitions can have far-reaching effects on the whole ecosystem (Lürling, 2003).

One emerging paradigm is that signals from eukaryotic-associated bacteria may drive multicellular complexity. *C. owczarzaki* can form aggregates under axenic conditions (Sebe-Pedros et al., 2013) and filamentous colonies in *S. rosetta* occur without bacteria (Dayel et al., 2011), but *S. rosetta* rosette colony formation is induced only in the presence of it's bacterial prey species (Dayel et al., 2011), which releases a specific sphingolipid signal that induces rosette formation (Alegado et al., 2012), perhaps enabling *S. rosetta* to feed more efficiently. Signals from microorganisms also profoundly affect the development of green seaweeds and land plants (Parniske, 2000; Matsuo et al., 2005; Marshall et al., 2006; Spoerner et al., 2012), although both can form multicellular structures in axenic conditions.

### “green” multicellularity: independent acquisitions

Acquisition of multicellularity underpinned the key transition of plants to land, but the mechanism(s) by which this occurred are unknown. Land plants evolved from algae (Figure 1A); algae have evolved multicellularity many times independently of land plants. In particular, the Chlorophyte algae include many “simple” multicellular species alongside morphologically complex green seaweeds (Niklas, 2014). Genomic information to define mechanisms underpinning “green” unicellular–multicellular transitions is lacking compared to the holozoan–animal comparative studies.

As with animals, it seems that the genetic toolkit for green multicellularity was present in unicellular ancestors (*Chlamydomonas*/*Volvox* genomes were discussed in Section Evolution of Unicellular–Multicellular Transitions: A Genetic Toolkit for Multicellularity?). Colonial “morphs” can be induced, or selected for, in usually unicellular microalgae e.g., *Chlamydomonas* (Lurling and Beekman, 2006; Ratcliff et al., 2013) and *Chlorella* (Boraas et al., 1998). Formation of the *Chlamydomonas* diploid zygote requires homologs of transcription factors involved in land plant meristem specification, implying that plant multicellular body plans may have evolved from algal sexual developmental mechanisms (Lee et al., 2008). In *Scenedesmus*, multicellularity correlated with reactive oxygen species production and activation of a kinase (Leflaive et al., 2008); redox state may also have been a driver of animal multicellularity (Blackstone, 2000).

It is technically difficult to study the unicellular–multicellular transition in most land plants at the molecular or genetic level. Embryos/gametes exist only transiently in the flowering plant life cycle, and are surrounded by multicellular tissues (Figure 1B). Early-evolving spore-bearing plants such as mosses and ferns are more experimentally tractable models for studying unicellular–multicellular transitions, as a unicell, the spore, can be isolated (and stored) prior to germination into multicellular structures (Figure 1C).

We believe that seaweeds are the best group of organisms in which to define the molecular changes involved in unicellular–multicellular transitions, as every seaweed has a unique life cycle that alternates between unicellular and multicellular stages, involving at least two separate unicellular–multicellular transitions: germination of spores into gametophytes, and germination of gametes and/or zygotes into sporophytes (Figure 1D). Thus, seaweeds represent exciting new systems in which unicellular–multicellular transitions can be studied with relative ease.

## Seaweeds as key systems to understand unicellular–multicellular transitions: understanding seaweed multicellularity will have economic importance

The emerging model systems *Ulva* (a green seaweed) and *Ectocarpus* (a brown seaweed) offer new ways to define the molecular basis of unicellular–multicellular transitions in complex systems. Relatively large numbers of *Ulva* unicells (spores or gametes) can be induced and isolated, far more easily than the spores of land plants, from more than one *Ulva* species (Wichard and Oertel, 2010; Vesty et al., 2015). It is this propensity for multicellular tissues to form unicells (which give rise to new multicellular structures) that leads to *Ulva* forming nuisance blooms: thus it is key to understand the basic biology of these processes. *Ulva* can now be grown in axenic laboratory-based culture, including *via* parthenogenesis (Wichard and Oertel, 2010; Spoerner et al., 2012), enabling a step-change in the methods we can use to understand seaweed biology and development. *Ectocarpus* has well-developed forward genetics, including a series of mutants (Le Bail et al., 2011; Cock et al., 2014), some of which are specifically altered in the spatial organization of the multicellular body [forming callus instead of polarized filaments: *gri* mutants (Le Bail et al., 2010)], or impaired maintenance of cell–cell adhesion: *bib* mutants (Charrier et al., in review)].

Many green, red and brown seaweeds are of profound economic importance, both positively as sources of food, fuel and useful chemicals, and negatively as biofoulers (Callow and Callow, 2011) or invasive species that are damaging to marine ecosystems (Smetacek and Zingone, 2013). Until now, seaweeds have been a neglected group of organisms for large-scale gene discovery, thus understanding seaweed growth and development lags behind our equivalent knowledge in land plants. Genome sequences are available for the red seaweeds (and food crops) *Pyropia* and *Chondrus* (Collen et al., 2013; Nakamura et al., 2013), and for *Ectocarpus* (Cock et al., 2010) with an *Ulva* genome-sequencing project underway. *Chondrus* is also an emerging macroalgal model system, taking ~2 years to complete its life cycle in the lab. Thus, the time is right to instigate a step-change in the understanding of seaweed biology, including the regulation of its unicellular–multicellular transitions. This will enable new knowledge to apply to seaweed culture (for food and fuel) and control (of blooms and biofouling).

## Wider perspectives on understanding seaweed multicellularity

Current data suggests that the genetic toolkit underpinning unicellular–multicellular transitions was present in unicellular ancestors, in both Unikonts and “green” organisms. Whether the toolkit is conserved between animals and plants is unknown. Using new “green” systems such as spore-plants, microalgae and seaweeds to define the molecular changes underpinning unicellular–multicellular transitions will shed new light on this question, and will also enable new understanding of how multicellular land plants evolved. Combined with the application of new basic biological knowledge to economically and environmentally important seaweed species, we are entering a new era of plant- and algal biology.

### Conflict of interest statement

The authors declare that the research was conducted in the absence of any commercial or financial relationships that could be construed as a potential conflict of interest.
